# Convenience, efficacy, safety, and durability of INSTI-based antiretroviral therapies: evidence from the Italian MaSTER cohort

**DOI:** 10.1186/s40001-023-01276-3

**Published:** 2023-08-18

**Authors:** Shannan N. Rich, Paola Nasta, Eugenia Quiros-Roldan, Paolo Fusco, Alice Tondinelli, Cecilia Costa, Chiara Fornabaio, Nicola Mazzini, Mattia Prosperi, Carlo Torti, Giampiero Carosi, the MaSTER cohort group

**Affiliations:** 1 Department of Epidemiology, College of Public Health and Health Professions and College of Medicine, University of Florida, Gainesville, FL USA; 2 University Division of Infectious and Tropical Diseases, University of Brescia and Brescia ASST Spedali Civili Hospital, Brescia, Italy; 3 Unit of Infectious and Tropical Diseases, Department of Medical and Surgical Sciences, ‘‘Magna Graecia’’ University, Catanzaro, Italy; 4 Fondazione Policlinico Universitario A. Gemelli IRCCS, Rome, Italy; 5 Infectious Diseases Unit, S. Maria Annunziata Hospital, Florence, Italy; 6 Infectious Diseases Unit, Cremona ASST Hospital, Cremona, Italy; 7 M.I.S.I. Foundation, Brescia, Italy

**Keywords:** HIV, Integrase inhibitors, Antiretroviral therapy, Survival analysis, MaSTER cohort

## Abstract

**Background:**

Integrase strand transferase inhibitors (INSTI), including raltegravir (RAL), elvitegravir (ELV), and dolutegravir (DTG), have demonstrated better efficacy and tolerability than other combination antiretroviral therapy (cART) classes in clinical trials; however, studies of sustainability of INSTI-containing therapy in the long-term are sparse. The purpose of this study was to provide an epidemiological overview comparing the outcome performance of different INSTI-based regimens longitudinally, including the metrics of efficacy, safety, convenience, and durability among a large, nationally representative cohort of persons living with HIV in Italy.

**Methods:**

We selected subjects in the MaSTER cohort (an Italian multicenter, hospital-based cohort established in the mid-1990s that currently has enrolled over 24,000 PLWH) who initiated an INSTI-based regimen either when naïve or following a regimen switch. Cox proportional hazards regression models were fitted to evaluate associations between therapy interruptions and age, sex, nationality, transmission risk group, viral suppression status, CD4 + T-cell count, diagnosis year, cART status (naïve or experienced), and hepatitis coinfection. Results were stratified by cART INSTI type.

**Results:**

There were 8173 participants who initiated an INSTI-based cART regimen in the MaSTER cohort between 2009 and 2017. The population was majority male (72.6%), of Italian nationality (88.6%), and cART-experienced (83.0%). Mean age was 49.7 (standard deviation: 13.9) years. In total, interruptions of the 1st INSTI-based treatment were recorded in 34% of cases. The most frequently cited reason for interruption among all three drug types was safety problems. In the survival analysis, past history of cART use was associated with higher hazards of interruption due to poor efficacy for all three drug types when compared to persons who were cART naïve. Non-viral suppression and CD4 + T-cell count < 200/mm^3^ at baseline were associated with higher hazards of interruption due to efficacy, safety, and durability reasons. Non-Italian nationality was linked to higher hazards of efficacy interruption for RAL and EVG. Age was negatively associated with interruption due to convenience and positively associated with interruption due to safety reasons. People who injects drugs (PWID) were associated with higher hazards of interruption due to convenience problems. Hepatitis coinfection was linked to higher hazards of interruption due to safety concerns for people receiving RAL.

**Conclusion:**

One-third of the population experienced an interruption of any drugs included in INSTI therapy in this study. The most frequent reason for interruption was safety concerns which accounted for one-fifth of interruptions among the full study population, mainly switched to DTG. The hazard for interruption was higher for low baseline CD4 + T-cell counts, higher baseline HIV-RNA, non-Italian nationality, older age, PWID and possible co-infections with hepatitis viruses. The risk ratio was higher for past history of cART use compared to persons who were cART naive, use of regimens containing 3 drugs compared to regimens containing 2 drugs. Durability worked in favor of DTG which appeared to perform better in this cohort compared to RAL and EVG, though length of follow-up was significantly shorter for DTG. These observational results need to be confirmed in further perspective studies with longer follow-up.

## Introduction

For individuals with HIV [with detectable viremia], the International Antiviral Society recommends a combination antiretroviral therapy (cART) of two nucleoside reverse transcriptase inhibitors (NRTIs) and one integrase strand transferase inhibitor (INSTI) as an optimal initial regimen unless otherwise indicated [1, 2]. Certain regimens may be contraindicated for some PLWH who suffer from clinical conditions such as cardiovascular, kidney, or liver diseases, opportunistic infections, or who are pregnant or planning to become pregnant, among other reasons [1, 2]. INSTIs including raltegravir (RAL), elvitegravir (ELV), and dolutegravir (DTG), have demonstrated better efficacy and tolerability than other cART classes in clinical trials [3–5]; however, studies of INSTI maintenance and indications for switching therapies in the long-term are sparse.

The purpose of this study was to evaluate the performance of cART INSTI-based regimens, stratified by INSTI type (RAL, ELV, and DTG), controlling for demographics, viro-immunological parameters, cART status (experienced versus naive), and 2-drug vs 3-drug regimens, with respect to the metrics of efficacy, safety and convenience, that together compounds the metric of durability of the INSTI-based regimen, among a large, nationally representative longitudinal cohort of people living with HIV (PLWH) in Italy. Data were obtained from the Italian MaSTER (Management Standardizzato di TErapia antiRetrovirale, translation: standardized management of antiretroviral therapy) cohort. The MaSTER cohort is a multicenter, hospital-based cohort established in the mid-1990s and currently has enrolled over 24,000 PLWH [6]. Data are available on enrolled persons’ medical, prescription, and laboratory records at regular time intervals (baseline, 6 months, and 12 months).

## Methods

### Inclusion criteria

Participants in the MaSTER cohort who initiated an INSTI-based regimen either at baseline (after diagnosis) or following a regimen switch between 2009 and 2017 were included in the study.

### Outcomes

Participants were followed until therapy interruption or until the end of the study in December 2017, whichever occurred first, apart from participants who were included in 2017, for whom a minimum follow-up of 12 months was guaranteed. We evaluated the performance of cART INSTI-based regimens, overall and stratified by INSTI type (RAL vs ELV vs DTG), using the metrics of efficacy, safety and convenience that together constituted the ‘‘durability’’ of the INSTI-based regimen. Interruption of an INSTI-based regimen for the metrics of efficacy was defined as individuals who had a virological failure (viral load level > 50 HIV-RNA copies/ml at least six months after initiating therapy). Interruption for the metrics of safety was defined as a laboratory alteration of grades 3–4 and/or clinical progression. Newly occurring grade 3 + laboratory alterations were defined using the following measurements [7]: aspartate transaminase (AST) > 260 units/L, alanine transaminase (ALT) > 235 units/L, bilirubin > 2.6 mg/dL, creatinine ≥ 1.9 mg/dL, calcium < 7 mg/dL, total cholesterol ≥ 275 mg/dL, glycemia > 250 mmol/L, triglycerides > 750 mg/dL and iron < 2 g/dL. Conditions considered to be clinical progressions of disease included a diagnosis of Acquired Immunodeficiency Syndrome (AIDS), cancer, cirrhosis defined as a FIB-4 ≥ 3.25 [8], an ischemic cardiovascular event, a renal event defined as eGFR (Estimated Glomerular Filtration Rate) < 89 ml/min, and any-cause death. Interruption for the metrics of convenience was defined as having HIV RNA > 50 copies/ml and a laboratory alteration (composite outcome of efficacy and safety). Interruption for the metrics of durability (considering it as the composite outcome of efficacy, safety and convenience) was defined as interruption regardless of tolerability, viral load level, laboratory alteration, or clinical progression.

### Statistical approach

Cox proportional hazards regression models were fitted to associate incidence of interruptions on INSTI therapies with demographic (age, sex, and nationality), enrollment period (cohort 2009–2011, cohort 2012–2014, cohort 2015–2017), and clinical factors [transmission risk factor (heterosexual, PWID, MSM, or other), diagnosis year, time between diagnosis and therapy, type of cART i.e. two- or three-drug regimen (2D/3D), cART status (naïve or experienced), INSTI type (DTG, EVG, or RAL), baseline CD4 + T-cell count (< 500 cells, 500 + cells) and viral load results (binary: suppressed at < 50 copies/ml or not suppressed), and history of positive hepatitis B surface antigen (HBsAg +) or hepatitis C antibodies (HCV). Additional stratified survival analyses were conducted by specific INSTI drug types. Analyses and data visualizations were performed in R statistical programming software using the following packages: survival [9], ggplot2 [10], survminer [11].

## Results

### Characteristics of study population

There were 8173 PLWH who initiated an INSTI-based cART regimen in the MaSTER cohort between 2009 and 2017. The population was majority male (72.6%), of Italian nationality (88.6%), with a past history of cART use (83.0%) and initiated on an INSTI-based regimen in the latest cohort (2015–2017, 72.6%) (Table 1). Mean age ranged from 48.4 (standard deviation [SD] = 17.2) to 50.6 years (SD = 14.4) for all 3 INSTI types. Median diagnosis year was 2005 [interquartile range (IQR): 1998–2013]. The majority of the population (69.9%) was virally suppressed (< 50 copies/mL) and had a CD4 + T-cell count above 500 cells/mm3 (54.0%) at baseline (Table 1).

**Table 1:** Characteristics of the full study population and of those receiving specific INSTI-based therapies

	Full population (N = 8173)	DTG (n = 3914)	EVG (n = 1331)	RAL (n = 2928)
Mean age (sd)	49.7 (13.9)	50.6 (14.4)	48.4 (17.2)	49.0 (11.4)
Age category				
< 50 years	4189 (51.3%)	1773 (45.3%)	789 (59.3%)	1627 (55.6%)
≥ 50 years	3984 (48.7%)	2141 (54.7%)	542 (40.7%)	1301 (44.4%)
Sex				
Female	2198 (27.4%)	1007 (26.3%)	330 (25.7%)	861 (29.6%)
Male	5819 (72.6%)	2823 (73.7%)	952 (74.3%)	2044 (70.4%)
Nationality				
Italian	6443 (88.6%)	3051 (88.1%)	914 (84.9%)	2478 (90.8%)
Non-Italian	827 (11.4%)	412 (11.9%)	163 (15.1%)	252 (9.2%)
Risk group				
Heterosexual	2929 (35.8%)	1406 (35.9%)	498 (37.4%)	1025 (35.0%)
PWID	2054 (25.1%)	890 (22.7%)	214 (16.1%)	950 (32.4%)
MSM	1637 (20.0%)	873 (22.3%)	344 (25.8%)	420 (14.3%)
Other	1553 (19.0%)	745 (19.0%)	275 (20.7%)	533 (18.2%)
Median diagnosis year (IQR)	2005 (1998–2013)	2006 (1998–2013)	2010 (2002–2015)	2002 (1998–2010)
Median viral load_Log10_ (IQR) Viral load at baseline	1.70 (1.70–2.39)	1.70 (1.70–1.94)	1.70 (1.70–3.13)	1.70 (1.70–2.78)
< 50	5712 (69.9%)	2877 (73.5%)	908 (68.2%)	1927 (65.8%)
50–9999	1211 (14.8%)	526 (13.4%)	172 (12.9%)	513 (17.5%)
> 10,000	1250 (15.3%)	511 (13.1%)	251 (18.9%)	488 (16.7%)
Median CD4 count (IQR) CD4 count at baseline	530 (320–770)	582 (364–821)	560 (354–761)	455 (267–684)
< 500 cells	3519 (46.0%)	1468 (39.9%)	527 (43.0%)	1524 (55.6%)
500 + cells	4123 (54.0%)	2208 (60.1%)	699 (57.0%)	1216 (44.4%)
Regimen				
2 drugs	2023 (24.8%)	977 (25.0%)	0 (0.0%)	1046 (35.7%)
3 drugs	6150 (75.2%)	2937 (75.0%)	1333 (100.0%)	1882 (64.3%)
cART status				
Naïve	1388 (17.0%)	650 (16.6%)	331 (24.9%)	407 (13.9%)
Experienced	6785 (83.0%)	3264 (83.4%)	1000 (75.1%)	2521 (86.1%)
Cohort				
2009–11	1134 (13.9%)	15 (0.4%)	0 (0.0%)	1119 (38.2%)
2012–14	1103 (13.5%)	24 (0.6%)	101 (7.6%)	978 (33.4%)
2015–17	5936 (72.6%)	3875 (99.0%)	1230 (92.4%)	831 (28.4%)
Hepatitis at baseline				
No	5435 (66.5%)	2726 (69.6%)	1017 (76.4%)	1692 (57.8%)
Yes	2738 (33.5%)	1188 (30.4%)	314 (23.6%)	1236 (42.2%)
Interruption for: efficacy	846 (10.4%)	316 (8.1%)	125 (9.4%)	405 (13.8%)
Convenience	822 (10.1%)	261 (6.7%)	96 (7.2%)	465 (15.9%)
Safety	1832 (22.4%)	458 (11.7%)	153 (11.5%)	1221 (41.7%)
Durability	2784 (34.1%)	746 (19.1%)	266 (20.0%)	1772 (60.5%)

*DTG* dolutegravir, *EVG* elvitegravir, *RAL* raltegravir, *sd* standard deviation, *PWID* people who inject drugs, *MSM* men who have sex with men, *IQR* interquartile range, *cART* combination antiretroviral therapy

**Fig. 1:**
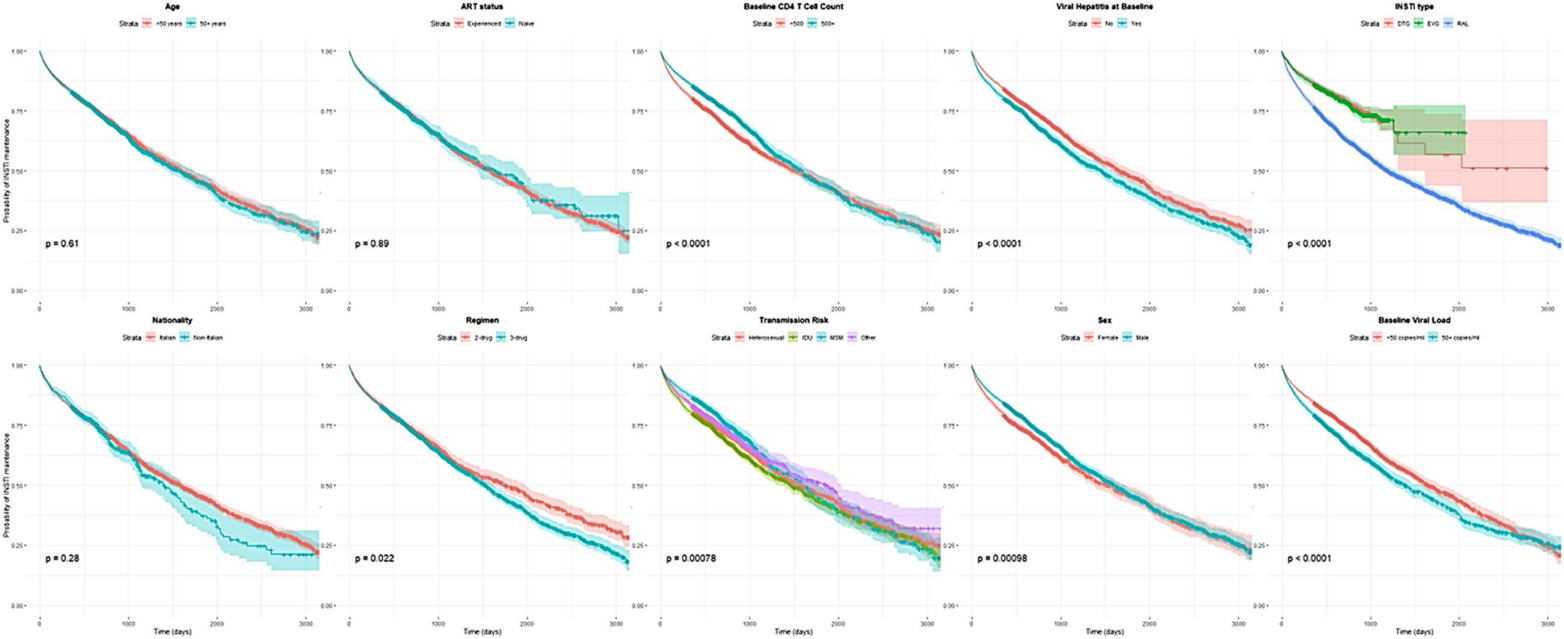
Kaplan Meier survival curves show the time to interruption of integrase strand transferase inhibitor (INSTI)-based antiretroviral therapies among persons with HIV enrolled in the Italian MaSTER cohort, between 2009 and 2017. Plots are stratified by characteristics of the full study population i.e.: regimen (2-drug vs 3-drug), INSTI drug type *DTG* dolutegravir, *EVG* elvitegravir, or *RAL* raltegravir, baseline CD4 count (< 200, 200–349, 350–499, 500 + cells), baseline viral load (< 50, 50–9999, 10,000 + copies) etc.

**Fig. 2:**
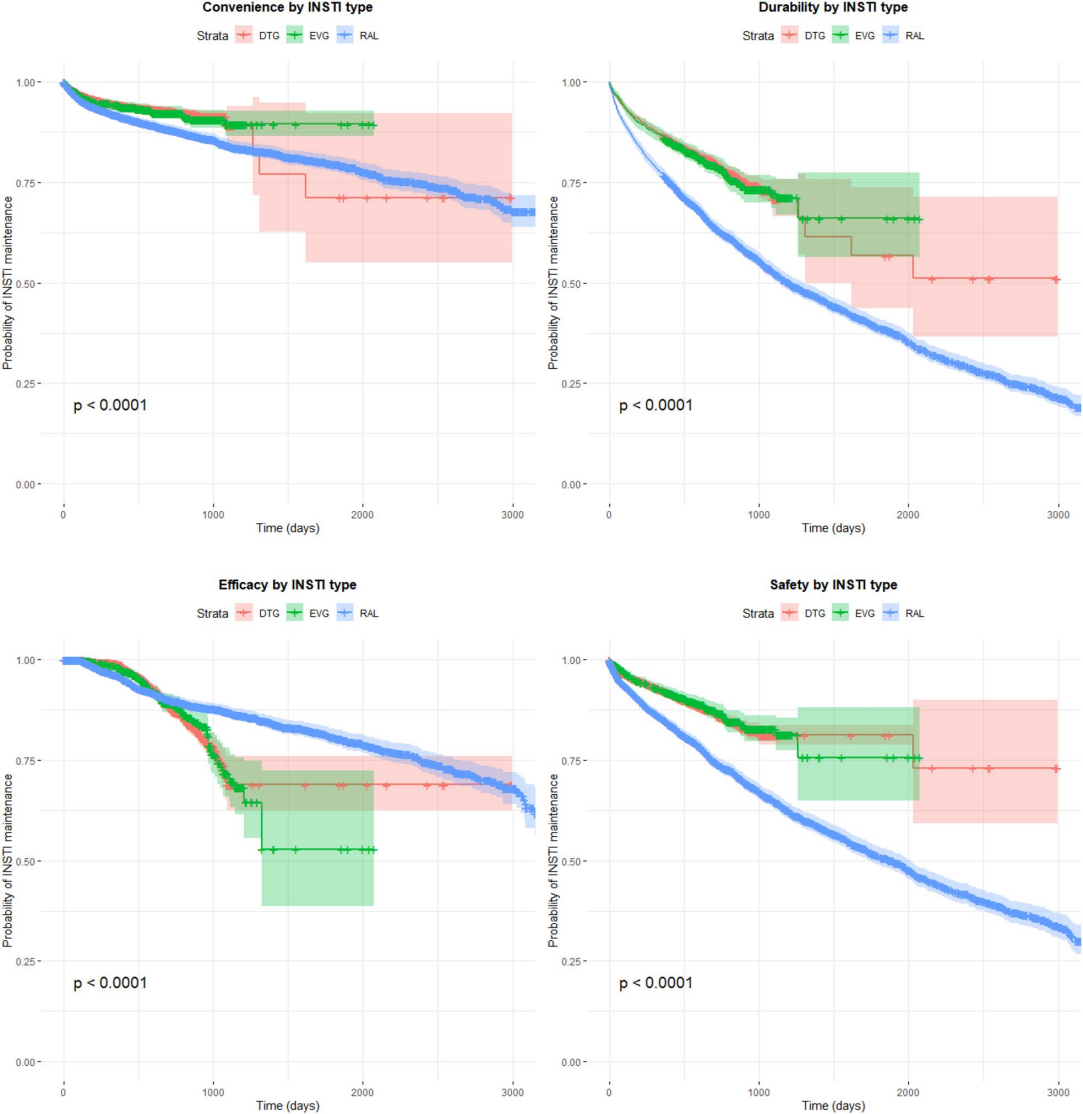
Kaplan Meier survival curves show the time to interruption of integrase strand transferase inhibitor (INSTI)-based antiretroviral therapies due to issues of convenience, durability, efficacy, and safety among persons with HIV enrolled in the Italian MaSTER cohort, between 2009 and 2017. Plots are stratified by INSTI-drug name: *DTG* dolutegravir, *ELV* elvitegravir, *RAL* raltegravir

The reported transmission risk factors were heterosexual (35.8%), PWID (25.1%), men who have sex with men (20.0%), or others (19.0%). Past or current hepatitis B or C coinfection was prevalent in 33.5% of the population (Table 1). The median time to interruption was 476 days in cART-experienced individuals and 293 in naïve individuals (Fig. 1) Interruptions linked to durability, representing the composite outcome, were most common (34.1%), followed by safety (22.4%), efficacy (10.4%), and convenience (10.1%) (Table 1), (Fig. 2).

A larger percentage of individuals aged 50 years and older were receiving DTG (54.7%), compared to the younger (< 50 years) population. Nearly all (99.0%) of DTG users initiated the therapy in the 2015–2017 cohort. A higher percentage of individuals with a hepatitis coinfection at baseline received RAL (42.2%). The median diagnosis year ranged considerably between drug groups (2006 [IQR: 1998–2013] for DTG, 2010 [IQR: 2002–2015] for EVG, and 2002 [IQR: 1998–2010] for RAL) (Table 1).

### Associations with interruption for the metrics of durability

Greater hazards of interruption for the metrics of durability were observed among individuals aged 50 years and over (hazards ratio [HR]: 1.14, 95% confidence interval [95%CI] 1.05–1.24), who were not virally suppressed at baseline (HR: 1.25, 95%CI 1.14–1.37), who were taking a 3-drug therapy versus a 2-drug therapy (HR: 1.21, 95%CI 1.10–1.32), who were positive for HCV or HBV at baseline (HR: 1.15, 95%CI 1.03–1.28), and who initiated therapy in more recent cohorts (HR: 1.78, 95%CI 1.49–2.13 in 2012–2014 and HR: 1.43, 95%CI 1.08–1.89 in 2015–2017) compared to the earlier cohort. Males had lower hazards of interruption compared to females (HR: 0.85, 95%CI 0.78–0.94). Further, increasing year of diagnosis and duration of time between diagnosis and therapy initiation were negatively associated with interruption (HR: 0.92, 95%CI 0.88–0.96 and HR: 0.91, 95%CI 0.87–0.95, respectively) (Table 2).

**Table 2:** Hazards of interruption due to the metrics analyzed

	Hazards of interruption (95% confidence interval) due to:			
	Efficacy	Convenience	Safety	Durability
Age				
< 50 years	Reference category	Reference category	Reference category	Reference category
≥ 50 years	0.88 (0.75;1.03)	0.65 (0.55;0.77)	1.51 (1.36;1.67)	1.14 (1.05;1.24)
Sex				
Female	Reference category	Reference category	Reference category	Reference category
Male	0.97 (0.82;1.16)	0.69 (0.58;0.83)	0.92 (0.82;1.04)	0.85 (0.78;0.94)
Nationality				
Italian	Reference category	Reference category	Reference category	Reference category
Non-Italian	1.22 (0.96;1.54)	1.27 (1.00;1.62)	0.91 (0.76;1.1)	1.07 (0.93;1.23)
Risk group				
Heterosexual	Reference category	Reference category	Reference category	Reference category
PWID	1.21 (0.95;1.54)	1.49 (1.16;1.91)	1.01 (0.87;1.18)	1.10 (0.97;1.25)
MSM	0.84 (0.65;1.08)	1.21 (0.95;1.54)	0.93 (0.79;1.08)	0.98 (0.86;1.11)
Other	1.02 (0.80;1.29)	0.90 (0.69;1.17)	0.93 (0.79;1.1)	0.92 (0.81;1.06)
Diagnosis year	1.25 (1.16;1.36)	0.91 (0.84;0.99)	0.91 (0.87;0.96)	0.92 (0.88;0.96)
Virally suppressed at baseline				
Yes	Reference category			
No	0.97 (0.79;1.21)	1.10 (0.91;1.32)	1.17 (1.05;1.32)	1.25 (1.14;1.37)
CD4 count at baseline				
< 500 cells	Reference category	Reference category	Reference category	Reference category
500 + cells	0.71 (0.60;0.84)	1.16 (0.98;1.37)	0.86 (0.78;0.96)	0.93 (0.86;1.02)
cART status				
Naïve	Reference category	Reference category	Reference category	Reference category
Experienced	2.05 (1.55;2.71)	0.77 (0.58;1.03)	1.16 (0.94;1.42)	1.09 (0.93;1.28)
Regimen				
2 drugs	Reference category	Reference category	Reference category	Reference category
3 drugs	0.98 (0.83;1.16)	1.17 (0.98;1.40)	1.23 (1.11;1.38)	1.21 (1.10;1.32)
Cohort				
2009–11	Reference category	Reference category	Reference category	Reference category
2012–14	1.09 (0.76;1.56)	1.66 (1.17;2.37)	1.83 (1.47;2.26)	1.78 (1.49;2.13)
2015–17	1.03 (0.61;1.73)	1.97 (1.16;3.34)	1.31 (0.94;1.84)	1.43 (1.08;1.89)
Time between diagnosis and therapy (days)	1.26 (1.16;1.37)	0.90 (0.83;0.98)	0.90 (0.86;0.95)	0.91 (0.87;0.95)
Hepatitis at baseline				
Not	Reference category	Reference category	Reference category	Reference category
Yes	0.97 (0.79;1.21)	1.04 (0.84;1.30)	1.19 (1.04;1.36)	1.15 (1.03;1.28)

*PWID* People who inject drugs, *MSM* men who have sex with men, *cART* combination antiretroviral therapy

### Associations with interruption for the metrics of efficacy

In the survival analysis, increasing year of diagnosis was associated with higher hazards of interruption for the metrics of efficacy (HR: 1.25, 95%CI 1.16–1.36), along with time between diagnosis and therapy (HR: 1.26, 95%CI 1.16–1.37), non-viral suppression at baseline (HR: 4.34, 95%CI 3.67–5.13), and past history of cART use (HR: 2.05, 95%CI 1.55–2.71) (Table 2).

### Associations with interruption for the metrics of convenience

Lower hazards of interruption for the metrics of convenience were observed among individuals aged 50 years and older compared to individuals < 50 years (HR: 0.65, 95%CI 0.55–0.77), males vs females (HR: 0.69, 95%CI 0.58–0.83), and a shorter duration of time between diagnosis and therapy initiation (HR: 0.90, 95%CI 0.83–0.98). In contrast, higher hazards of interruption were observed among PWID vs heterosexual risk group (HR: 1.49, 95%CI 1.16–1.91) and among individuals initiating INSTI-based therapies in more recent cohorts (HR: 1.66, 95%CI 1.17–2.37 in 2012–2014 and HR: 1.97, 95%CI 1.16–3.34 in 2015–2017) compared to the earliest cohort (2009–2011) (Table 2).

### Associations with interruption for the metrics of safety

Higher hazards of interruption due to safety concerns were observed among individuals ages 50 years and older (HR: 1.51, 95%CI 1.36–1.67), those not virally suppressed at baseline (HR: 1.17, 95%CI 1.05;1.32), taking a 3-drug vs a 2-drug therapy (HR: 1.23, 95%CI 1.11–1.38), individuals in the 2012–2014 cohort compared to the earlier cohort (HR: 1.83, 95%CI 1.47–2.26), and those with a history of hepatis (HR:1.19, 95%CI 1.04–1.36). Increasing year of diagnosis, a CD4 count above 500 cells, and time between diagnosis and therapy were negatively associated with the hazards of interruption (HR: 0.91, 95%CI 0.87–0.96, HR: 0.86, 95%CI 0.78–0.96, and HR: 0.90, 95%CI 0.86–0.95, respectively) (Table 2).

### Associations with interruption by strata of therapy type

Within the survival analysis stratified by therapy type (DTG, RAL, or EVG), higher hazards of interruption were observed among individuals aged 50 years and older (compared to individuals < 50 years) due to safety concerns for DTG and RAL (HR: 1.92, 95%CI 1.52–2:43 for DTG; HR: 1.40, 95% CI 1.24–1.58 for RAL), and due to durability issues for RAL only (HR: 1.13, 95%CI 1.02–1.25). This age group had lower hazards of interruption due to convenience for DTG (HR: 0.59, 95% CI 0.44–0.79) and RAL (HR: 0.67, 95%CI 0.54–0.83). We observed that males had lower hazards of interruption for the metrics of convenience when on DTG (HR: 0.56, 95%CI 0.40–0.76), for the metrics of safety when on EVG (HR: 0.64, 95%CI 0.42–0.99), and for the metrics of durability when on either DTG (HR: 0.76, 95%CI 0.63–0.91) or EVG (HR: 0.67, 95%CI 0.47–0.94). Individuals of non-Italian nationality had higher hazards of interruption for the metrics of efficacy on RAL and EVG (HR: 1.79, 95%CI 1.29–2.49 for RAL and HR: 1.77, 95%CI 1.03–3.06 for EVG) and lower hazards of interruption for the metrics of safety on DTG (HR: 0.62, 95%CI 0.41–0.94).

Risk group was associated with higher hazards of interruption for the metrics of convenience among PWID versus heterosexual individuals taking DTG (HR: 1.62, 95%CI 1.01–2.62) or RAL (HR: 1.49, 95%CI 1.09–2.04). Diagnosis year was positively associated with interruptions only among RAL users for the metrics of convenience (HR: 1.03, 95%CI 1.01–1.05) and durability (HR: 1.02, 95%CI 1.01–1.03). Being non-virally suppressed at baseline was associated with higher hazards of interruption for the metrics of efficacy for all drug types (HR: 3.97, 95%CI 3.01–5.24 for DTG; HR: 4.29, 95%CI 3.39–5.44 for RAL, and HR: 3.44, 95%CI 2.18–5.41 for EVG), convenience for EVG (HR: 1.94, 95%CI 1.06–3.57), safety for EVG (HR: 1.60, 95%CI 1.04–2.47), and durability for RAL (HR: 1.17, 95%CI 1.04–1.30) and EVG (HR: 1.90, 95%CI 1.36–2.66). Having a CD4 + T-cell count equal or greater than 500 cells/mm^3^ was associated with lower hazards of interruption for the metrics of efficacy on DTG (HR: 0.55, 95%CI 0.42–0.73) and with higher hazards of interruption due to convenience (HR: 1.32, 95%CI 1.07–1.63). Individuals with a past history of cART use had higher hazards of interruption for the metrics of efficacy for all drug types (HR: 1.89, 95%CI 1.23–2.92 on DTG; HR: 1.89, 95%CI 1.26–2.84 on RAL, and HR: 2.57, 95%CI 1.21–5.45 on EVG). Being diagnosed with HCV or HBV infections at baseline was associated with higher hazards of interruption for the metrics of safety on RAL (HR: 1.23, 95%CI 1.05–1.44) (Table 3).

**Table 3:** Hazards of interruption stratified by INSTI type. *Ref. cat*.: Reference category

	Hazards of interruption (95% confidence interval) due to:											
	Efficacy			Convenience			Safety			Durability		
	DTG	RAL	EVG	DTG	RAL	EVG	DTG	RAL	EVG	DTG	RAL	EVG
Age												
< 50 years	Ref. cat	Ref. cat	Ref. cat	Ref. cat	Ref. cat	Ref. cat	Ref. cat	Ref. cat	Ref. cat	Ref. cat	Referent	Referent
≥ 50 years	1.00 (0.76;1.31)	1.06 (0.85;1.32)	1.04 (0.66;1.64)	0.59 (0.44;0.79)	0.67 (0.54;0.83)	0.71 (0.40;1.25)	1.92 (1.52;2.43)	1.40 (1.24;1.58)	1.44 (0.97;2.13)	1.19 (1.00;1.41)	1.13 (1.02;1.25)	1.08 (0.79;1.47)
Sex												
Female	Ref. cat	Ref. cat	Ref. cat	Ref. cat	Ref. cat	Ref. cat	Ref. cat	Ref. cat	Ref. cat	Ref. cat	Referent	Referent
Male	1.09 (0.80;1.48)	0.92 (0.72;1.17)	0.74 (0.46;1.19)	0.56 (0.40;0.76)	0.79 (0.63;1.00)	0.73 (0.39;1.35)	0.88 (0.69;1.11)	1.01 (0.87;1.16)	0.64 (0.42;0.99)	0.76 (0.63;0.91)	0.94 (0.84;1.06)	0.67 (0.47;0.94)
Nationality												
Italian	Ref. cat	Ref. cat	Ref. cat	Ref. cat	Ref. cat	Ref. cat	Ref. cat	Ref. cat	Ref. cat	Ref. cat	Ref. cat	Ref. cat
Non-Italian	0.88 (0.57;1.35)	1.79 (1.29;2.49)	1.77 (1.03;3.06)	1.39 (0.93;2.07)	1.24 (0.89;1.73)	1.14 (0.56;2.30)	0.62 (0.41;0.94)	1.04 (0.83;1.31)	0.81 (0.45;1.46)	0.88 (0.67;1.17)	1.15 (0.96;1.37)	1.06 (0.70;1.61)
Risk group												
Heterosexual	Ref. cat	Ref. cat	Ref. cat	Ref. cat	Ref. cat	Ref. cat	Ref. cat	Ref. cat	Ref. cat	Ref. cat	Ref. cat	Ref. cat
PWID	1.32 (0.86;2.03)	1.21 (0.88;1.66)	1.76 (0.91;3.40)	1.62 (1.01;2.62)	1.49 (1.09;2.04)	1.33 (0.59;3.00)	1.22 (0.87;1.7)	0.93 (0.78;1.11)	1.05 (0.59;1.89)	1.31 (1.00;1.71)	1.03 (0.88;1.20)	1.08 (0.68;1.72)
MSM	0.75 (0.50;1.14)	0.95 (0.67;1.36)	0.82 (0.42;1.61)	1.21 (0.79;1.85)	1.19 (0.86;1.65)	1.52 (0.77;3.03)	0.92 (0.68;1.24)	0.89 (0.73;1.08)	1.38 (0.82;2.34)	0.97 (0.76;1.23)	0.93 (0.79;1.09)	1.41 (0.95;2.11)
Other	1.28 (0.89;1.84)	0.77 (0.53;1.11)	1.03 (0.58;1.85)	0.90 (0.57;1.43)	0.96 (0.67;1.35)	0.57 (0.23;1.43)	0.76 (0.54;1.06)	0.95 (0.78;1.16)	1.21 (0.71;2.04)	0.87 (0.67;1.13)	0.93 (0.79;1.10)	0.90 (0.58;1.39)
Diagnosis year	1.01 (0.99;1.04)	1.02 (1.00;1.05)	0.97 (0.93;1.01)	1.01 (0.99;1.04)	1.03 (1.01;1.05)	1.01 (0.96;1.06)	1.02 (1.00;1.04)	1.02 (1.00;1.03)	1.01 (0.98;1.05)	1.01 (1.00;1.03)	1.02 (1.01;1.03)	1.01 (0.98;1.04)
Not virally suppressed at baseline	3.97 (3.01;5.24)	4.29 (3.39;5.44)	3.44 (2.18;5.41)	1.01 (0.70;1.47)	0.99 (0.79;1.25)	1.94 (1.06;3.57)	1.17 (0.90;1.52)	1.11 (0.97;1.26)	1.60 (1.04;2.47)	1.19 (0.97;1.47)	1.17 (1.04;1.30)	1.90 (1.36;2.66)
CD4 count at baseline												
< 500 cells	Ref. cat	Ref. cat	Ref. cat	Ref. cat	Ref. cat	Ref. cat	Ref. cat	Ref. cat	Ref. cat	Ref. cat	Ref. cat	Ref. cat
500 + cells	0.55 (0.42;0.73)	1.05 (0.83;1.32)	0.72 (0.46;1.13)	1.15 (0.85;1.56)	1.32 (1.07;1.63)	0.90 (0.53;1.54)	0.88 (0.71;1.09)	0.92 (0.81;1.05)	0.75 (0.51;1.10)	0.93 (0.79;1.10)	1.03 (0.92;1.14)	0.77 (0.57;1.04)
cART status												
Naïve	Ref. cat	Ref. cat	Ref. cat	Ref. cat	Ref. cat	Ref. cat	Ref. cat	Ref. cat	Ref. cat	Ref. cat	Ref. cat	Ref. cat
Experienced	1.89 (1.23;2.92)	1.89 (1.26;2.84)	2.57 (1.21;5.45)	0.84 (0.48;1.47)	0.80 (0.55;1.17)	0.92 (0.42;2.00)	1.11 (0.70;1.75)	1.10 (0.86;1.40)	1.65 (0.84;3.21)	1.10 (0.78;1.55)	1.06 (0.87;1.29)	1.34 (0.83;2.15)
Positive hepatitis diagnosis at baseline	0.91 (0.62;1.32)	1.13 (0.85;1.50)	0.82 (0.45;1.50)	1.19 (0.78;1.81)	0.89 (0.67;1.17)	1.60 (0.80;3.21)	0.93 (0.70;1.26)	1.23 (1.05;1.44)	1.44 (0.88;2.33)	1.01 (0.80;1.28)	1.13 (0.99;1.29)	1.47 (1.00;2.17)

*INSTI* integrase strand transfer inhibitor, *Ref. cat*. reference category, *DTG* dolutegravir, *EVG* elvitegravir, *RAL* raltegravir, *PWID* people who inject drugs, *MSM* men who have sex with men, *cART* combination antiretroviral therapy

## Discussion

In this study, we evaluated the performance of cART INSTI-based regimens, overall and stratified by INSTI type (RAL vs ELV vs DTG), using the metrics of efficacy, safety and convenience that together constituted the ‘’durability’’ of the INSTI-based regimen, in a large Italian cohort of 8173 people.

We observed that durability of the INSTI-based therapy was shorter in people aged 50 years and older, especially because of higher hazards of interruption due to safety concerns for dolutegravir and raltegravir, while for the metrics of convenience we observed a protective effect by older age with an hazard ratio of interruption significantly < 1. Although INSTI regimens may represent a convenient strategy in older PLWH, our findings appear to reinforce the recommendation to further optimize drug regimens in older populations with frailty phenotypes and with a greater risk of drug-drug interactions because of polypharmacy [12–16]. This recommendation is reinforced by the observations that PLWH aged 55 years or older had poorer clnical outcomes after adjusting for the last recorded CD4 + T-cell counts [17]

A lower risk of interruption for the metrics of durability of the INSTI-based regimens was also associated with a longer duration between HIV diagnosis and start of INSTI-based therapy (considering it as the sum of time from diagnosis of HIV infection to cART initiation and the time from cART initiation to start of INSTI regimens) and this effect appeared to be driven by convenience and safety problems. By contrast, for the metrics of efficacy, increasing time between HIV diagnosis and therapy predicted an increased risk of interruption. In addition, individuals who were not virally suppressed prior to initiating the new INSTI therapy had a higher hazard of interruption particularly due to efficacy problems compared to individuals who were virally suppressed at baseline. Also we found that durability of the INSTI-based regimen was lower in experienced participants than in naïve participants because of their higher hazard of interruption for the metrics of efficacy, which was true for all three INSTI-drugs. Overall, these findings suggest that INSTI based regimens were used effectively as simplification treatment even in PLWH with a longer duration of HIV infection (from diagnosis to INSTI start), but these PLWH should be carefully monitored for HIV RNA given a greater risk of interruption because of virological failure. Therefore, starting with an INSTI-based therapy as soon as possible may preserve durability of these regimens [18]. Interestingly, a protective effect on the durability of the INSTI-combined regimen was seen in PLWH having CD4 + T-cell counts ≥ 500/mm^3^, especially in those on dolutegravir, underlying the importance of greater CD4 + T-cell counts even in PLWH prescribed such well tolerated and effective drugs [19].

Another finding of the study was that taking a 3-drug compared with a 2-drug regimen was associated with greater risk of stopping because of durability reasons related to the metrics of safety and convenience. This may be due to a reduced pill burden resulting in better adherence of PLWH to HIV treatment and sustained HIV-RNA control [20]. Moreover, it was demonstrated that “dual regimens” may improve safety profiles by excluding the use of drugs associated with specific toxicities such as renal impairment and increased levels of lipids [21]. These findings were in agreement with the results of a study of the Italian ODOACRE cohort, however made exclusively on virologically suppressed PLWH, in which 2-drug regimens showed an efficacy similar to 3-drug regimens but with better tolerability [22]. However, the use of dual regimens has to be tailored to patient profiles since a systematic review and meta-analysis showed that “dual regimens” may even be associated with a greater risk of failure and selection of resistance-associated mutations at 96 weeks of follow-up in some circumstances [23].

Individuals of non-Italian nationality did not report significant greater risk of interrupting INSTI for any of the metrics considered. However, when we explored the risk of interruption by individual INSTI, a significant lower risk of interruption was found in favor of dolutegravir for the metric of safety, while a significant greater risk of interruption of either raltegravir or elvitegravir was found for the metric of efficacy. It is therefore possible that a higher significant barrier of dolutegravir against the emergence of HIV drug resistance allowed an effective use of this drug in the context of simpler and better tolerated regimens compared to the other INSTI, optimizing both safety and efficacy particularly in migrants [24, 25].

PWID reported higher hazard of interruption of the INSTI-based treatment compared to heterosexual individuals particularly for the metric of convenience related to the use of DTG and RAL. It is known that active intravenous drug use is a major obstacle to effective cART in PLWH [26–28], so they require more intensive follow-up and additional medical support to remain engaged in cART treatment program over the long term.

Individuals enrolled in cohorts 2012–2014 and 2015–2017 had higher hazards of interruption concerning durability compared with individuals enrolled in the earliest cohort (2009–2011), in particular for the metric of convenience. This may indicate that there was an increasing tendency to switch for simplification reasons overtime given the availability of DTG as new drugs with better toxicity/tolerability and simpler to take because of co-formulation.

Positive hepatitis B surface antigen (HBsAg +) or hepatitis C antibodies (HCV) were only associated with higher hazards of interruption for the metrics of durability in the overall population, mainly due to safety concerns among those receiving RAL. Since there were no significant signals of increased risk of hepatotoxicity due to RAL in the literature [29–34], it is difficult to explain this finding. However, RAL was the first INSTI introduced into clinical practice when the burden of patients with chronic hepatitis related to HCV co-infections at advanced stage of liver fibrosis was predominant since the majority of these patients did not have the opportunity to take the new direct-acting antivirals achieving HCV eradication at that time. Likewise, the increased risk associated with RAL may be due to patient characteristics, more than to hepatotoxicity of the drug by itself.

Lastly, our data highlighted that durability of the INSTI-based regimen worked in favour of DTG which appeared to perform better in this cohort compared to RAL and EVG. This finding appears to be consistent with the results of a systematic review and network meta-analysis made on naïve participants that concluded that PLWH receiving DTG had lower odds of discontinuing therapy by week 96 compared to protease inhibitors, efavirenz, RAL or EVG/cobicistat [35]**.** Similarly, data from SCOLTA cohort showed that compared to DTG, treatment with protease inhibitors or EVG/cobicistat were associated with an increased risk of interruption [36].

This work is affected by the limitations typical of any observational-retrospective studies. First, it is possible that participants included more recently in the cohort were characterized by better conditions as an effect of the “selection-of-the fittest” bias as already discussed for the higher hazards of interruption for the metrics of durability in the overall population, mainly due to safety concerns among those receiving RAL. Second, despite the fact that a minimum follow-up of 12 months was guaranteed for any participants in order to control for the “immortality bias” which could favor drugs introduced into clinical practice more recently (therefore with a lower probability to register the outcomes of interest for a shorter time of observation), we cannot exclude that for PLWH who started an INSTI-based treatment in the latest cohort 2015–2017 (most of them based on DTG), the short follow-up precluded the achievement of the predefined outcomes of the study. Third, when participants were divided by type of INSTI, the sample size in each group was small, making this analysis only exploratory. For instance, the variable “diagnosis year” showed a slightly protective effect in the overall cohort particularly for the metrics of safety and convenience, which is consistent with the availability of better drugs in the modern eras. However, when the performance of specific INSTI was evaluated, a slightly harmful effect for both metrics was observed, possibly indicating an unreliable control for this variable in the final multivariable model. For these reasons, a prospective evaluation of DTG based regimens was already performed in the MaSTER cohort, showing that DTG based regimens were maintained in 84.4% participants with a modest incidence of interruptions mostly due to cART simplification strategies [37]. Fourth, since our work was mostly epidemiological in nature, we did not assess time-varying variables of clinical interest which should be addressed in future analyses. Lastly, despite the fact that in this work participant’s enrollment stopped six years ago, when DTG was used less frequently in clinical practice compared to recent years, this limitation was mitigated by a fairly great number of PLWH using a DTG based regimen in our cohort (3914/8173 participants). We also recognize that today in clinical practice there is a tendency to abandon using RAL and ELV for safety and simplification reasons, in favour of the newest available INSTI like DTG, bictegravir (BIC) and cabotegravir (the last two not included in the analysis). Notwithstanding the above limitations, we feel that the large sample size (total participant number was 8173), the “real-life” conditions, and the multi-dimensional evaluation with several outcomes, may provide interesting observations on the way INSTI was used and how the regimens containing this drug class should be optimized, providing a starting point for future studies on the newest INSTI-regimens, including BIC and cabotegravir (CAB).

## Conclusions

In conclusion, interruptions of the 1st INSTI-based treatment were recorded in 34% of cases, with decreasing frequency in the subsequent three-year periods. The most frequent reason for interruption was a modest frequency due to toxicity which accounted for one-fifth of interruption among the full study population, mainly switched to DTG. The hazard for interruption was higher for low baseline CD4 + T-cell counts, higher baseline HIV-RNA, non-Italian nationality, older age, PWID and possible co-infections with hepatitis viruses. The risk ratio was higher for past history of cART use compared to persons who were cART naive, 3D regimens compared to 2D regimens. In addition, even though this analysis is limited by small sample size in the treatment subgroups, DTG appeared to perform better in this cohort compared to RAL and EVG with respect to the metrics of durability. More powerful results (greater sample size, longer follow-up) are needed on the newest INSTI-regimens, including BIC and CAB, considering also the emerging adverse events reported, such as increasing weight gain and early cardiovascular events [38, 39]. Finally, it is important to study interruptions of individual drugs in the combination regimens which may be due to drug specific adverse events which were not accounted for in this work, such as osteoporosis and fragility fractures [40], neuropsychiatric adverse events [41], malignancies [42] or the risk of drug-drug interactions [43].

## Data Availability

The data can be made available upon reasonable request by submitting a request to the Malattie Infettive e Salute Internazionale (MISI, translation: Infectious Diseases and International Health Foundation) Foundation in Brescia, Italy.

## Abbreviations

INSTI: Integrase strand transferase inhibitors
RAL: Raltegravir
ELV: Elvitegravir
DTG: Dolutegravir
BIC: Bictegravir
CAB: Cabotegravir
cART: Combination antiretroviral therapy
HIV: Human immunodeficiency virus
CD4: Cluster of differentiation-4
PWID: People who injects drugs
NRTIs: Reverse transcriptase inhibitors
PLWH: People living with HIV
MaSTER: Management Standardizzato di TErapia antiRetrovirale, translation: standardized management of antiretroviral therapy
AST: Aspartate transaminase
ALT: Alanine transaminase
AIDS: Acquired immunodeficiency syndrome
FIB-4: Fibrosis-4
eGFR: Estimated glomerular filtration rate
MSM: Men who have sex with men
2D: Two drug
3D: Three drug
HBV: Hepatitis B virus
HCV: Hepatitis C virus
HBsAg: Hepatitis B surface antigen
SD: Standard deviation
IQR: Interquartile range
HR: Hazard ratio
CI: Confidence interval
